# Single-slab 3D double inversion recovery for magnetic resonance brain imaging in clinically healthy dogs

**DOI:** 10.3389/fvets.2023.1156870

**Published:** 2023-07-17

**Authors:** Miseong Je, Sunho Yang, Dongjae Lee, Jihye Choi, Junghee Yoon

**Affiliations:** College of Veterinary Medicine and the Research Institute for Veterinary Science, Seoul National University, Seoul, Republic of Korea

**Keywords:** brain, dog, double inversion recovery, gray matter, magnetic resonance imaging, white matter

## Abstract

**Introduction:**

In veterinary medicine, magnetic resonance imaging (MRI) is widely utilized for brain imaging. But the complex structures of brain tissues can give rise to artifacts such as partial volume averaging in conventional sequences. To address this issue, several studies about double inversion recovery (DIR) sequences have been conducted in human medicine. However, published clinical studies about brain MRI using DIR sequences in dogs are currently lacking. The purpose of this study was to evaluate the magnetic resonance features of single-slab 3D DIR sequences in the normal canine brain.

**Methods:**

Five healthy Beagle dogs were examined and the following pulse sequences were acquired for each: (1) spin-echo T2-weighted (T2W), (2) fluid attenuated inversion recovery (FLAIR), (3) gray matter (GM) selective, and (4) white matter (WM) selective single-slab 3D DIR sequence. For qualitative analysis, the distinction between gray and white matter of the cerebral cortex, presence and severity of the image artifacts were assessed for each pulse sequence. In addition, reconstructed images of single-slab 3D DIR sequences were qualitatively evaluated. For quantitative analysis, contrast ratios (CRs), signal-to-noise ratios (SNRs), and contrast-to-noise ratios (CNRs) of the GM, WM and cerebrospinal fluid (CSF) were measured for each pulse sequence.

**Results and Discussion:**

GM selective 3D DIR was superior to T2W and FLAIR in delineating the boundaries between GM and WM in the overall brain area. Whereas WM selective 3D DIR provided better gray-white matter distinction of the cerebral cortex than T2W and FLAIR at the level of the medulla oblongata, where T2W and FLAIR images exhibited severe partial volume averaging artifacts. In general, the 3D DIR images demonstrated fewer artifacts compared to other sequences, and the reconstructed sagittal and dorsal images of these sequences maintained same spatial resolution as the original transverse images without any image degradation. Both gray and white matter selective 3D DIR sequences effectively suppressed unwanted signals, thereby providing high contrast between gray and white matter. Findings from this study could serve as a foundation for further studies on DIR sequences for the evaluation of brain diseases in dogs.

## 1. Introduction

Given the high soft tissue resolution and utility of specialized sequences, MRI is the preferred modality for brain imaging (1, 2). However, it should be considered that complicatedly folded brain structure may cause some imaging artifacts. One of the most common artifacts encountered in clinical situation is partial volume averaging artifact, which easily occurs at the curved interface between different brain structures, and it can be manifested as pseudolesions (3). Conversely, true lesions might be obscured by partial volume averaging, especially in lesions adjacent to cerebrospinal fluid (CSF) (4). Consequently, errors in interpreting MR images could occur with conventional MRI sequences such as T2-weighted (T2W) and fluid attenuated inversion recovery (FLAIR) (5, 6). To complement these sequences, double inversion recovery (DIR) sequence has been introduced in several human studies (5–7). In the DIR sequence, two different 180° inversion radiofrequency (RF) pulses are applied before a classic fast or turbo spin-echo acquisition, allowing two different signals to be nulled simultaneously (8). Thus, DIR can selectively depict gray matter (GM) by suppressing white matter (WM) and CSF signals, or WM by suppressing GM and CSF signals, and was known for providing markedly high contrast resolution between gray and white matter with superior delineation in human patients (4, 8). Especially, GM selective DIR, which selectively images GM by suppressing the signals from both WM and CSF, has been reported to provide better conspicuity of cortical and subcortical lesions in various central nervous system (CNS) diseases than other MR techniques (9).

DIR imaging was initially introduced using two-dimensional (2D) multislice sequences at 1.5-Tesla (T) (4, 8, 10). However, due to the complex morphology of the brain, three-dimensional (3D) MR imaging with high spatial resolution is preferred over 2D MR imaging. Some studies have reported that a multislab 3D DIR sequence improves the spatial resolution and detection of intracortical lesions, but the presence of flow artifacts and signal intensity differences between slabs cannot be resolved with multislab 3D sequence (6, 11, 12). On the other hand, single-slab 3D DIR sequence has a long echo train and variable flip angles for refocusing RF pulses, which covers whole brain with high quality and without flow artifacts from CSF or blood (12). Consequently, multislab 3D DIR sequence has been replaced by single-slab 3D DIR sequence (9).

In human medicine, DIR sequences have been widely used to evaluate various neurologic diseases including multiple sclerosis, epilepsy, and Alzheimer’s disease, all of which exhibit cortical lesions or changes in cortical thickness or volume (9, 13, 14). However, to the authors’ knowledge, published clinical studies about brain MRI using DIR sequences in dogs are currently lacking. The purpose of this study was to evaluate the magnetic resonance features of single-slab 3D DIR sequences in the normal canine brain. Authors hypothesized that single-slab 3D DIR sequences, either gray or white matter selective, would provide higher tissue contrast resolution than conventional T2W and FLAIR sequences, and would identify the exact boundary between brain tissues with fewer artifacts in clinically healthy dogs.

## 2. Materials and methods

### 2.1. Study design and description of dogs

This investigation was a prospective, methods comparison, exploratory study. Five purpose-bred healthy, intact male Beagle dogs were used in the study. The median age was 4 years (range, 4–5 years) and the median weight was 14 kg (range, 12.8–15.5 kg). For each dog, the screening tests including physical and neurological examination, thoracic radiographs and abdominal ultrasonographic examination were done prior to the procedures by a veterinarian (MJ) with 2 years of radiology experience. The dogs are owned by College of Veterinary Medicine, Seoul National University and all the procedures performed in the study were approved by the Seoul National University Institutional Animal Care and Use Committees (SNU-220807-1).

### 2.2. Data recording: brain MRI protocol

Magnetic resonance imaging examinations were performed with the dogs under general anesthesia, using a 1.5-T MR scanner (GE Signa, 1.5T, GE healthcare). Anesthetic protocols were the following: medetomidine (0.01 mg/kg IM, Domitor, Zoetis) was used for premedication, alfaxalone (2.0 mg/kg IV, Alfaxan, Jurox Pty Ltd) for induction, and isoflurane (Ifran, Hana Pharm) for maintenance. Noninvasive blood pressure, oxygen saturation, heart rate, body temperature, and end-tidal carbon dioxide concentration were monitored during the anesthesia.

The dogs were positioned in sternal recumbency on an 8-channel phased-array knee coil. Spin-echo T2W, FLAIR, GM and WM selective single-slab 3D DIR images were obtained in transverse plane from each dog. Table 1 details the parameters that were used for each sequence. To reduce the acquisition time for 3D DIR sequences, number of excitations (NEX) was adjusted from 4 to 2. The acquisition time for each sequence was automatically recorded. After the examination, complications related to the anesthesia were monitored for 5 days in each dog.

**Table 1:** Pulse sequence parameters used for brain magnetic resonance imaging in dogs.

Parameter	T2W	FLAIR	GM selective 3D DIR	WM selective 3D DIR
TR (ms)	5,904	8,000	5,000	5,000
TE (ms)	80	100	80	80
TI (ms)	–	2,433	2,562/605	2,290/419
ETL	20	20	140	140
ST (mm)	3	3	2	2
Locs per slab	–	–	50	50
FOV (mm)	200	200	200	200
Matrix	288 × 224	256 × 192	176 × 176	176 × 176
NEX	4	2	2	2
Acquisition time (min:sec)	4:49	8:1	5:24	5:23

TR, repetition time; TE, echo time; TI, inversion time; ETL, echo train length; ST, slice thickness; FOV, field-of-view; NEX, number of excitations; T2W, T2-weighted; FLAIR, fluid attenuated inversion recovery; DIR, double inversion recovery; 3D, three-dimensional; GM, gray matter; WM, white matter.

### 2.3. Data analysis

The qualitative and quantitative analysis of brain images were conducted using a DICOM viewer software (RadiAnt DICOM Viewer, version 4.6.9, free evaluation edition, Medixant, Poznan, Poland). The qualitative analysis was performed by one veterinarian (MJ) with 2 years of radiology experience, under the supervision of a veterinarian (JY) with more than 30 years of diagnostic imaging expertise. The quantitative measurements were performed by three other veterinarians (MJ, SY, and DL) with 1–2 years of radiology experience. Because of obvious signal differences, the readers could not be entirely blinded to the type of the sequences.

In qualitative assessment, the images of each sequence were compared in the same transverse planes. The images were evaluated in six different anatomical regions; at the level of the frontal lobe, optic chiasm, interthalamic adhesion, mesencephalic aqueduct, pons, and medulla oblongata. For each plane, distinction between gray and white matter of the cerebral cortex was assessed using a four-point scale: (1) not visualized at all; (2) poorly visualized but possible to detect; (3) clearly visualized with blurry junction; and (4) clearly visualized with sharp junction. In addition, the readers assessed the image quality according to the artifacts, including motion, partial volume averaging, and flow artifacts. This evaluation was also conducted using a four-point scale: (1) “poor”: pronounced artifacts present, limiting diagnostic capability; (2) “fair”: artifacts do not prevent diagnostic capability but degrade image quality; (3) “good”: minor artifacts present without significant impact on image quality; and (4) “excellent”: no artifacts observed. Meanwhile, transverse images of gray and white matter selective 3D DIR sequences were reconstructed using multiplanar reconstruction, then sagittal and dorsal planes were assessed to determine the presence of signal intensity variations along the slab direction.

**Figure 1:**
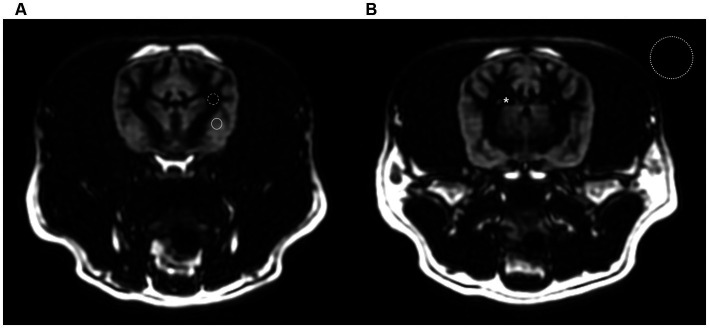
Transverse gray matter selective three-dimensional double inversion recovery images at the level of the optic chiasm **(A)** and interthalamic adhesion **(B)** in a dog. The region of interest (ROI) of gray and white matter were chosen from the thickest and most uniform area possible (solid line and dashed line). The ROI for CSF measurement was taken within the lateral ventricles (asterisk). Standard deviation of background was measured in a background air containing no image artifacts (dotted line).

In quantitative assessment, contrast ratios (CRs), signal-to-noise ratios (SNRs), and contrast-to-noise ratios (CNRs) of the GM, WM and CSF were calculated as follows:

$$CR_{tissue\;1,2}=\frac{SI_{tissue\;1}-SI_{tissue\;2}}{SI_{tissue\;1}+SI_{tissue\;2}}$$

$$SNR_{tissue}=\frac{SI_{tissue}}{SD_{air}}$$

$$CNR_{tissue\;1,2}=\frac{SI_{tissue\;1}-SI_{tissue\;2}}{SD_{air}}$$

where SI_tissue_ is the mean tissue signal intensity measured in arbitrary units; SD_air_ the standard deviation of background signal. The CRs and CNRs are calculated using the absolute value because a positive or a negative contrast between the two tissues is considered to be equal (15). All values are given as mean ± SD. Signal intensities of individual types of tissues were assessed by region of interest (ROI) measurements with the ROI placed identically on each sequence (Figure 1). The gray and white matter ROI was chosen from the thickest and most uniform area possible, the CSF measurement was taken within the lateral ventricles. Standard deviation (SD) of background was determined by measuring the SD of the pixel intensities in a background air containing no image artifacts.

### 2.4. Statistical analysis

All statistical analysis was conducted by one author with diagnostic imaging expertise and statistical training (MJ) using commercially available software (SPSS statistical program, IBM SPSS Statistics 25, IBM Corporation, NY). The ordinal data were presented as medians and ranges, while the continuous data were presented as means and SD. All the parameters were compared using the Kruskal-Wallis *H* test and *post hoc* test with Bonferroni correction. Interobserver agreements between the three observers were assessed using the intraclass correlation coefficient (ICC) test. Variables with *P*-values of <0.05 were considered statistically significant.

## 3. Results

Brain MR images were attained with each sequence in all dogs without any anesthetic complications. No significant abnormalities were identified in brain MR images of all dogs. The total scan time was between 25 and 30 min in each dog. The acquisition time of each sequence is presented in Table 1.

**Figure 2:**
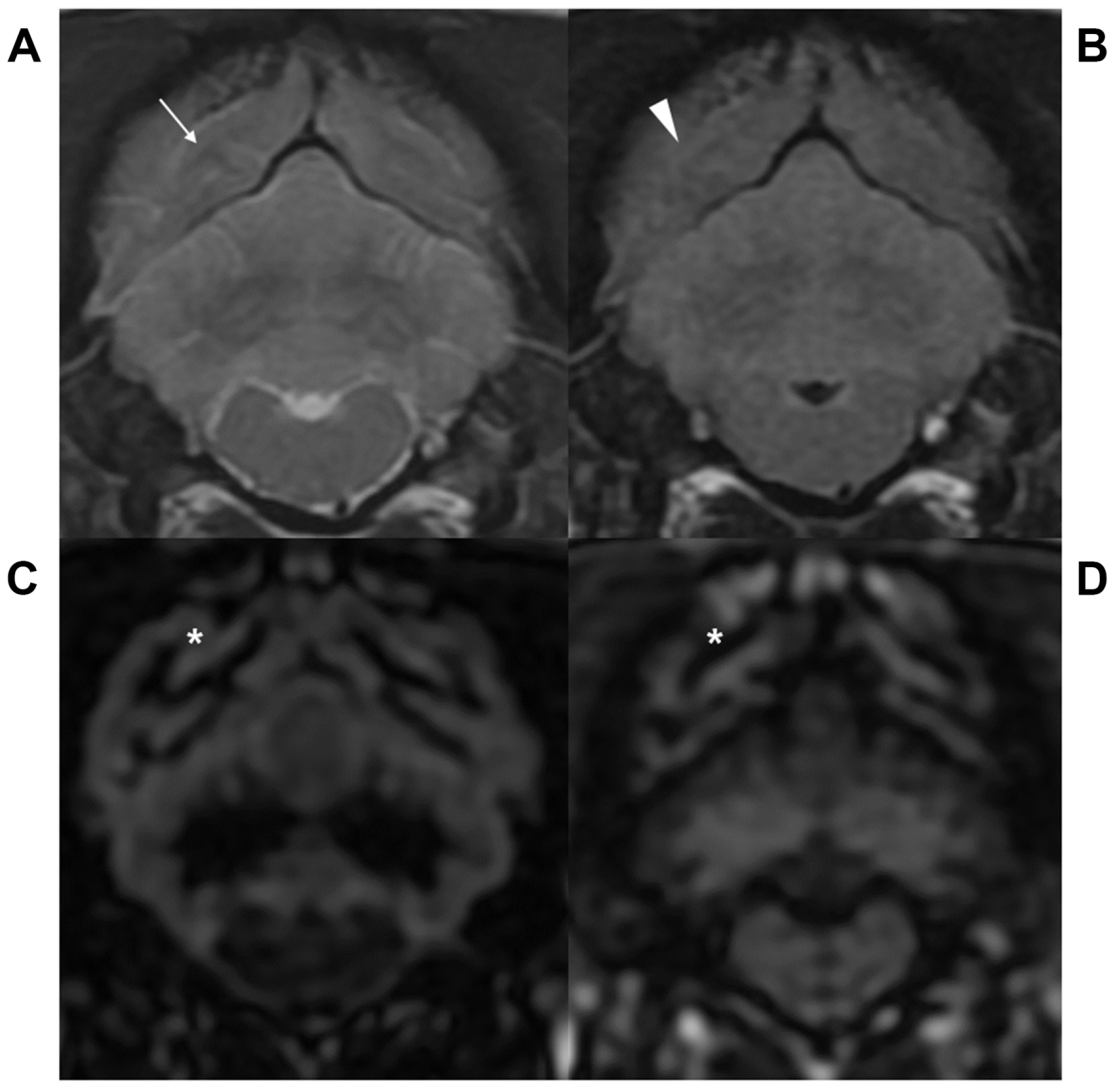
Transverse T2-weighted (T2W, **A**), fluid attenuated inversion recovery (FLAIR, **B**), gray matter selective three-dimensional double inversion recovery (GM selective 3D DIR, **C**), and white matter selective three-dimensional double inversion recovery (WM selective 3D DIR, **D**) images at the level of the medulla oblongata in a dog. T2W and FLAIR images showed blurry gray-white matter distinction (arrow and arrowhead), while GM and WM selective 3D DIR images clearly visualized the border of gray and white matter (asterisk) at the level of the medulla oblongata.

The score of gray-white matter distinction is displayed for each pulse sequence and each location (Table 2). In all six locations from the frontal lobe to the medulla oblongata level, gray-white matter distinction of the cerebral cortex was more clearly visualized on GM selective 3D DIR than T2W and FLAIR. GM selective 3D DIR also had statistically higher distinction score than WM selective 3D DIR at the level of the mesencephalic aqueduct and pons. WM selective 3D DIR did not have significant difference with T2W and FLAIR from the frontal lobe to the pons level, but showed significantly higher distinction score than T2W and FLAIR at the level of the medulla oblongata. T2W and FLAIR tended to blur gray-white matter distinction of the occipital lobe at the medulla oblongata level, and in three of five dogs, the border of gray and white matter of the occipital cortex was even not visible at all in FLAIR images at the level of the medulla oblongata. On the other hand, both GM and WM selective 3D DIR sequences clearly visualized gray-white matter distinction in that region (Figure 2). FLAIR generally showed poor gray-white matter distinction of the cerebral cortex except at the interthalamic adhesion level, and the distinction was particularly poor at the medulla oblongata level.

**Table 2:** Comparison of MR sequences for gray-white matter distinction score at six different locations.

	T2W [median (range)]	FLAIR [median (range)]	GM selective 3D DIR [median (range)]	WM selective 3D DIR [median (range)]	*P*-value
Frontal lobe	3 (0)^a^	2 (1.0)^b^	4 (0)^a, b^	3 (1.0)	0.002
Optic chiasm	3 (0)^a^	2 (1.0)^b^	4 (0)^a, b^	3 (1.0)	0.002
Interthalamic- adhesion	3 (0)^a^	3 (1.0)^b^	4 (0)^a, b^	3 (1.0)	0.002
Mesencephalic- aqueduct	3 (0)^a^	2 (1.0)^b^	4 (0)^a, b, c^	3 (1.0)^c^	0.001
Pons	3 (1.0)^a^	2 (0)^b^	4 (0)^a, b, c^	3 (1.0)^c^	0.002
Medulla- oblongata	2 (0)^a, b^	1 (1.0)^c, d^	4 (1.0)^a, c^	3 (0)^b, d^	0.001

T2W, T2-weighted; FLAIR, fluid attenuated inversion recovery; DIR, double inversion recovery; 3D, three-dimensional; GM, gray matter; WM, white matter.

^a, b, c, d^ Within a row, the same superscript indicated statistically significant differences between two groups using a Bonferroni correction (significance level of *P*-value < 0.0083).

**Figure 3:**
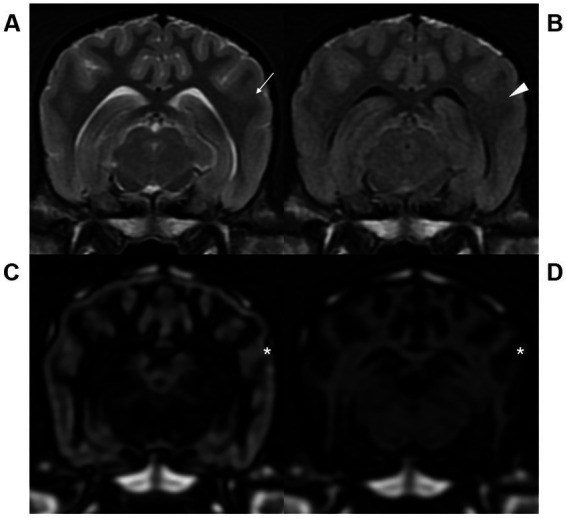
Transverse T2-weighted (T2W, **A**), fluid attenuated inversion recovery (FLAIR, **B**), gray matter selective three-dimensional double inversion recovery (GM selective 3D DIR, **C**), and white matter selective three-dimensional double inversion recovery (WM selective 3D DIR, **D**) images at the level of the mesencephalic aqueduct in a dog. Partial volume averaging artifacts at the gray-white matter junctions were more severe in T2W (arrow) and FLAIR (arrowhead) than GM and WM selective 3D DIR (asterisk) images, resulting in blurred gray-white matter distinction.

The image quality according to the artifacts are displayed for each pulse sequence (Table 3). Motion and flow artifacts were absent, regardless of the sequence in all dogs. On the other hand, partial volume averaging artifacts were detected in all sequences. These artifacts were usually identified at the narrow sulci and gyri region. The severity of partial volume averaging artifacts was significantly lower at 3D DIR images, compared to T2W and FLAIR. In the images of conventional sequences, it was found that the gray-white matter junctions were blurred due to severe artifacts (Figure 3).

**Table 3:** Comparison of MR sequences for image quality according to the artifacts.

	T2W [median (range)]	FLAIR [median (range)]	GM selective 3D DIR [median (range)]	WM selective 3D DIR [median (range)]	*P*-value
Motion artifacts	4 (0)	4 (0)	4 (0)	4 (0)	1.000
Flow artifacts	4 (0)	4 (0)	4 (0)	4 (0)	1.000
Partial volume averaging artifacts	2 (1.0) ^a, b^	1 (1.0) ^c, d^	3 (0) ^a, c^	3 (0) ^b, d^	0.001

T2W, T2-weighted; FLAIR, fluid attenuated inversion recovery; DIR, double inversion recovery; 3D, three-dimensional; GM, gray matter; WM, white matter.

^a, b, c, d^ Within a row, the same superscript indicated statistically significant differences between two groups using a Bonferroni correction (significance level of *P*-value < 0.0083).

**Figure 4:**
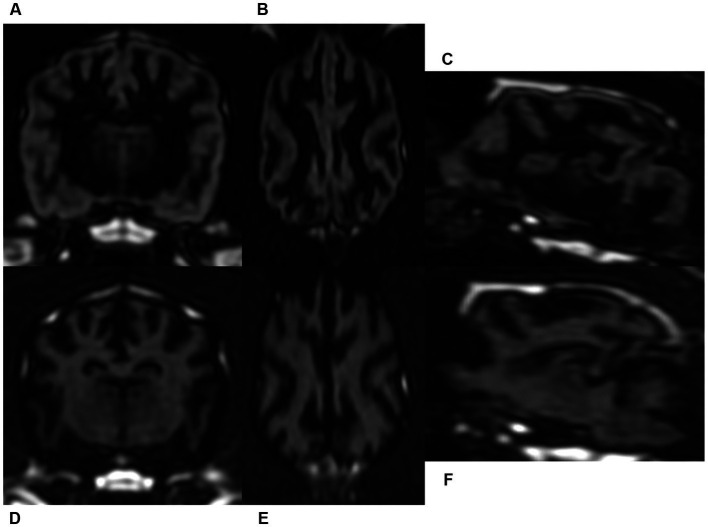
Gray matter selective three-dimensional double inversion recovery (GM selective 3D DIR, **A–C**) and white matter selective three-dimensional double inversion recovery (WM selective 3D DIR, **D–F**) images in a dog. Because of the nearly isotrophic resolution, reconstructed dorsal and sagittal images of GM selective 3D DIR **(B,C)** and WM selective 3D DIR **(E,F)** had identical image quality with the original transverse magnetic resonance images **(A,D)**.

By the single-slab excitation, which simultaneously excites the entire slab, 3D DIR images had homogeneous signal intensity along the slab direction. Thus, the reconstructed sagittal and dorsal images of GM and WM selective 3D DIR sequences had same spatial resolution as the original transverse images, without any visible image degradation (Figure 4).

For the measurements of quantitative parameters including CRs, SNRs, and CNRs, the intraclass correlation among the three readers showed excellent interobserver agreement (Table 4). The mean and SD for CR, SNR, and CNR of the GM, WM and CSF are displayed for each pulse sequence (Table 5). CR_GM-WM_ was markedly higher for GM and WM selective 3D DIR than T2W and FLAIR, and FLAIR had the lowest CR_GM-WM_ than other three sequences. CNR_GM-WM_ of GM and WM selective 3D DIR sequences were significantly higher than FLAIR. Although the mean values of CNR_GM-WM_ of both 3D DIRs were measured to be higher than T2W, differences were not statistically significant. There were no significant differences in CR_GM-WM_ and CNR_GM-WM_ between GM and WM selective 3D DIR. CR_GM-CSF_ of GM selective DIR was significantly higher than T2W, but there was no significant difference with FLAIR. CR_WM-CSF_ was significantly higher in WM selective DIR than both T2W and FLAIR. CNR_GM-CSF_ of GM selective DIR and CNR_WM-CSF_ of WM selective DIR have no statistical differences with T2W and FLAIR. No significant difference was found between sequences for SNR_GM_ except WM selective DIR, which suppressed GM signals. Likewise, no significant difference was found between sequences for SNR_WM_ except GM selective DIR, which suppressed WM signals.

**Table 4:** Intraclass correlation coefficient values for interobserver reliability of quantitative analysis.

Evaluation factors		ICC	95% confidence interval
CR	GM-WM	0.998	0.996–0.999
	GM-CSF	0.987	0.972–0.994
	WM-CSF	0.992	0.984–0.997
SNR	GM	0.940	0.875–0.975
	WM	0.952	0.899–0.979
	CSF	0.979	0.955–0.991
CNR	GM-WM	0.987	0.973–0.995
	GM-CSF	0.986	0.970–0.994
	WM-CSF	0.984	0.967–0.993

CR, contrast ratio; SNR, signal to noise ratio; CNR, contrast to noise ratio; GM, gray matter; WM, white matter; CSF, cerebrospinal fluid; ICC, intraclass correlation coefficient.

**Table 5:** Comparison of MR sequences for contrast between brain tissues.

	Ratio	T2W (mean ± SD)	FLAIR (mean ± SD)	GM selective DIR (mean ± SD)	WM selective DIR (mean ± SD)	*P*-value
GM	SNR	183.28 ± 43.383^a^	122.22 ± 32.306^b^	137.04 ± 54.595^c^	17.95 ± 4.712^a, b,c^	0.004
WM	SNR	114.27 ± 26.308^a^	83.77 ± 24.002^b^	15.01 ± 6.413^a, b, c^	146.43 ± 46.560^c^	0.004
CSF	SNR	315.05 ± 81.312^a, b, c^	21.30 ± 5.609^a^	16.00 ± 7.239^b^	19.90 ± 4.693^c^	0.007
GM-WM	CR	0.23 ± 0.018^a, b^	0.19 ± 0.020^a, b^	0.80 ± 0.042^a^	0.77 ± 0.044^b^	0.001
	CNR	69.02 ± 17.952^a^	38.45 ± 9.305^a, b, c^	122.03 ± 49.147^b^	128.48 ± 43.405^c^	0.002
GM-CSF	CR	0.26 ± 0.028^a, b^	0.70 ± 0.047^a^	0.79 ± 0.030^b^	0.10 ± 0.052^a, b^	0.001
	CNR	131.77 ± 40.783^a^	100.91 ± 27.695^b^	121.03 ± 47.701^c^	3.87 ± 2.199^a, b, c^	0.009
WM-CSF	CR	0.47 ± 0.011^a^	0.59 ± 0.064^a^	0.12 ± 0.076^a^	0.75 ± 0.043^a^	0.000
	CNR	200.78 ± 55.178^a^	62.46 ± 19.417^a^	3.45 ± 1.897^a, b^	126.53 ± 42.889^b^	0.001

SNR, signal to noise ratio; CR, contrast ratio; CNR, contrast to noise ratio; T2W, T2-weighted; FLAIR, fluid attenuated inversion recovery; DIR, double inversion recovery; 3D, three-dimensional; GM, gray matter; WM, white matter.

^a, b, c^ Within a row, the same superscript indicated statistically significant differences between two groups using a Bonferroni correction (significance level of *P*-value < 0.0083).

## 4. Discussion

Based on the author’s literature review, this is the first published study of the utility of 3D DIR sequences for brain imaging in clinically healthy dogs. In this study, we compared the brain images obtained by single-slab 3D DIR sequences with those of conventional sequences, T2W and FLAIR, qualitatively and quantitatively.

Our findings demonstrated that GM selective 3D DIR is superior to conventional T2W and FLAIR in delineating the boundaries between gray and white matter of the cerebral cortex in all six locations from the frontal lobe to the medullar oblongata level. These results are consistent with previous human studies, which have shown that GM selective DIR provides superior gray-white differentiation compared to T2W or FLAIR (4, 8). Whereas WM selective 3D DIR had no significant difference from the conventional sequences in visualization of gray-white matter distinction of the cerebral cortex, from the frontal lobe to the pons level. However, at the level of the medulla oblongata, where T2W and FLAIR images showed severe blurring of the gray-white matter junction of the cerebral cortex, not only GM selective 3D DIR but also WM selective 3D DIR provided much better visualization of the boundaries between gray and white matter. The narrow and tapered shape of the cerebral parenchyma at the level of the medulla oblongata resulted in severe partial volume averaging artifacts in conventional sequences. However, the utilization of two 3D DIR sequences with thin continuous slices effectively reduced the impacts of partial volume averaging artifacts, thereby resulting in improved distinction between gray and white matter of the cerebral cortex at the level of the medulla oblongata.

In a previous study, it has been reported that nonselective excitation of the single-slab 3D DIR can effectively prevent the occurrence of artifacts from blood flow or CSF pulsation (12). In our study, artifacts such as motion or flow artifacts were not visually apparent on 3D DIR images, except for mild partial volume averaging artifacts. But motion and flow artifacts were not visible in T2W and FLAIR images as well. In terms of the severity of artifacts, the only significant difference between conventional sequences and two 3D DIR sequences was observed in relation to partial volume averaging artifacts. The image quality of both two 3D DIR sequences was significantly less affected by partial volume averaging artifacts than T2W and FLAIR, because 3D DIR sequences obtained volumetric data and effectively overcame the artifacts.

An additional advantage of 3D DIR sequences is the isotrophic resolution, which enables the original images to be reconstructed in any orientation with same spatial resolution. This characteristic eliminates the need to repeat MR examinations with the same region in different planes.

In quantitative analysis, GM selective 3D DIR had significantly higher CR_GM-WM_ than other conventional sequences, T2W and FLAIR. In addition, GM selective 3D DIR had higher mean value of CR_GM-CSF_ than T2W and FLAIR, but showed no statistical difference with FLAIR, a sequence that suppresses the CSF signal. WM selective 3D DIR showed significantly higher CR_GM-WM_ and CR_WM-CSF_ than other conventional sequences. These results revealed that good degrees of unwanted signal suppression were achieved with both two 3D DIR sequences.

CNR, a parameter which takes into account the background noise, is regarded as clinically the most relevant parameter, and the ability to detect brain anatomy depends on CNR (16, 17). In our study, both GM and WM selective 3D DIR had significantly higher CNR_GM-WM_ than FLAIR. It suggests that both two 3D DIR sequences provide better contrast resolution between gray and white matter than FLAIR. In addition, the mean values of CNR_GM-WM_ of two 3D DIR sequences were higher than that of T2W, but differences were statistically non-significant. FLAIR showed significantly the lowest CR_GM-WM_ and CNR_GM-WM_, which suggests that the ability of gray-white matter differentiation would be the worst. CNR_GM-CSF_ of GM selective 3D DIR and CNR_WM-CSF_ of WM selective 3D DIR had no significant differences with other sequences, and even T2W had higher mean values of CNR_GM-CSF_ and CNR_WM-CSF_ than 3D DIR sequences. Therefore, it should be noted that 3D DIR sequences may not be superior for delineating the boundary between CSF regions such as ventricles or subarachnoid space and brain parenchyma. In this aspect, it is unlikely that 3D DIR sequences will completely replace conventional sequences. However, they can serve as a valuable supplement, particularly in the assessment of the gray-white matter junction.

Previous studies have stated that long scan time of DIR sequences may limit their routine clinical use (4, 8). In our current study, the parameters of 3D DIR sequences were adjusted for the acquisition time to be clinically acceptable. During the initial examination conducted on a Beagle dog, it was found that reducing the number of excitations (NEX) from 4 to 2 did not result in a noticeable difference in image quality. As a result, the scan time was reduced by the half and both GM and WM selective 3D DIR acquired shorter scan time (5 min 24 s in GM selective 3D DIR and 5 min 23 s in WM selective 3D DIR) than FLAIR (8 min 1 s). We assumed that adjustment of the parameters for reducing the scan time might lead to a substantial loss of SNR. But in our study, SNR_GM_ of GM selective 3D DIR and SNR_WM_ of WM selective 3D DIR had no statistical difference with other sequences. This finding differs from previous studies that reported relatively low SNRs for DIR sequences (4, 12).

Our study had some limitations. First, the dogs in the study were considered as normal based on only few MR sequences, physical and neurological examination and absence of clinical signs. To reduce anesthetic time, a complete MRI protocol including pre- and post-contrast T1-weighted (T1W) sequences or CSF analysis could not be conducted. Second, the signal intensities of each brain tissue were measured in arbitrary units without the use of a phantom for reference. Since the signal intensity is dependent on several factors such as imaging parameters, scanner settings, and acquisition techniques, the values obtained are not directly calibrated to a specific physical quantity. Third, the assessment of the clinical feasibility of 3D DIR sequences was limited as clinical patients with brain lesions were not included. The parameters measured in this study were not lesion-related values, but rather aimed to evaluate the contrast between brain tissues within each sequence. Therefore, in order to assess the lesion conspicuity in 3D DIR sequences, additional parameters should be evaluated in further studies.

Several CNS diseases in human are known to cause changes in GM, and numerous studies have reported the utility of 3D DIR sequences in evaluating these diseases (11). Likewise, in veterinary medicine, there are diseases that cause changes in GM, and the representative one is epilepsy (18). A study found the reduction of GM in dogs with idiopathic and structural epilepsy, by using voxel based morphometry (VBM) analysis (18). For VBM to function properly, the MRI image should have good contrast between the different brain tissues. However, the tissue distinction is not accurate especially in the sulcus region where the contrast between GM and adjacent WM can be reduced. Another problem is that the voxels containing several tissues may not be interpreted accurately, because the VBM model assumes that each voxel contains only one type of tissue (19). Considering the characteristics of 3D DIR sequences, which provide high contrast between gray and white matter with less partial volume averaging, the utilization of 3D DIR images for VBM analysis could potentially resolve these problems. In addition, 3D DIR sequences are expected to provide superior conspicuity of brain lesions located at the gray-white matter junction, and the diagnostic efficacy of 3D DIR sequences for brain lesions such as meningoencephalitis should be studied further.

In conclusion, both GM and WM selective single-slab 3D DIR sequences provided higher contrast between gray and white matter with fewer imaging artifacts than conventional sequences, including T2W and FLAIR. Therefore, the addition of a single-slab 3D DIR sequences to a standard MR brain imaging protocol could enhance the depiction of anatomical contrast. This study can also provide valuable background data for further studies about DIR sequences for evaluating brain diseases in dogs, and further studies are warranted using dogs with brain lesions.

## Data availability statement

The original contributions presented in the study are included in the article/supplementary material, further inquiries can be directed to the corresponding author.

## Ethics statement

The animal study was reviewed and approved by Seoul National University Institutional Animal Care and Use Committees (SNU-220807-1).

## Author contributions

MJ, JC, and JY contributed to the conception and design of the study. MJ, SY, and DL contributed to data acquisition. MJ and JY performed the data interpretation and drafted the manuscript. MJ performed the statistical data analysis and the interpretation. All authors revised the manuscript and gave their final approval.

## Conflict of interest

The authors declare that the research was conducted in the absence of any commercial or financial relationships that could be construed as a potential conflict of interest.
